# Combined therapy of pulsed radiofrequency and nerve block in postherpetic neuralgia patients: a randomized clinical trial

**DOI:** 10.7717/peerj.4852

**Published:** 2018-06-04

**Authors:** Dan Li, Guohua Sun, Hanzhe Sun, Yanjuan Wang, Zhiping Wang, Jianping Yang

**Affiliations:** 1 Department of Anesthesiology, Wuxi People’s Hospital, Wuxi, China; 2 Department of Anesthesiology, The First Hospital Affiliated to Soochow University, Suzhou, China

**Keywords:** Nerve block therapy, Pulsed radiofrequency, Postherpetic neuralgia

## Abstract

Caused by viral infection, postherpetic neuralgia (PHN) is the most common chronic neuropathic pain. Various treatment modalities such as early use of nerve block therapy (NBT) and pulsed radiofrequency (PRF) have been studied in reducing pain, however, no consistent success was achieved in all the patients treated with single regimen. The combined therapy of PRF and NBT with different targeting mechanism are of interest and remains to be determined. Here we investigated the combined effects of pulsed radiofrequency (PRF) with nerve block therapy (NBT) in PHN patients in a prospective randomized clinical trial. Sixty PHN patients were divided into four groups (*n* = 15 each): the conventional puncture group (group CP), the nerve block therapy group (group NB), the PRF group (group PRF), and the combined treatment group (PRF combined with nerve block therapy (group CT). To evaluate the extent of remission of hyperalgesia, we recorded the visual analogue scale (VAS) scores during cotton swab reaction before and after treatment and in the resting and active pain states. In addition, blood samples were collected and plasma cytokine and neuropeptides such as interleukin-6 (IL-6), substance P (SP), and β-endorphin (β-EP) were measured by enzyme-linked immunosorbent assay (ELISA) at the admission (basic state), before the operation, and at 12 h postoperatively. The number of adverse events (nausea, vomiting, constipation, puncture point hemorrhage, swelling and redness) within 12 h of the treatment were also documented. Our results showed that VAS scores during cotton swab reaction decreased after treatment in all patients (*p* < 0.05). Compared to group CP, plasma IL-6 and SP levels decreased (*p* < 0.05) and β-EP levels increased (*p* < 0.05) in groups NB, PRF, and CT. There were no significant differences in adverse events among groups (*p* > 0.05). We found that PRF in combination with NBT increased β-EP levels and decreased plasma IL-6 and SP, thereby alleviating pain and hyperalgesia in PHN patients. Taken together, our data suggest combined therapy of PRF and NBT is effective and safe for PHN patients.

## Introduction

Postherpetic neuralgia (PHN) is the most common chronic nerve pain caused by chickenpox (herpes zoster) viral infection. PHN leads to burning pain that lasts long after the skin rash disappear in some patients. There are limited pain management options because the underlying mechanisms remains unclear. Recent studies showed that traditional oral drugs, nerve block therapy (NBT) and pulsed radiofrequency (PRF) can shorten pain duration in some patients (Johnson & Rice, 2014; Kim et al., 2017; Shi & Wu, 2016). PHN can be severe and debilitating in some cases. However, no single treatment modality reduces pain for all patients with consistent success. Combinational treatments are needed in many PHN cases and similarly in chronic cervical radicular pain (McCrary, Severson & Tyring, 1999; Wang et al., 2017; Xiao et al., 2015).

Sensory abnormalities expressed in PHN patients includes dysesthesia (unpleasant and abnormal sensation), allodynia (painful response to normally innocuous stimuli) or hyperalgesia (heightened pain response) (Mallick-Searle, Snodgrass & Brant, 2016). Several generic questionnaires are available for assessment of pain intensity: unidimensional pain questionnaires (Visual Analog Scale and Numeric Rating Scale) and multidimensional pain questionnaires (Short-form McGill Pain Questionnaire, Chronic Pain Grade Scale, and Short Form-36 Bodily Pain Scale) (Johnson, 2005). In our study, light touch with a cotton swab is applied to assess the sensitivity to touch in the affected area. Pain intensity and quality is assessed using VAS score, a unidimensional measure of pain intensity widely used in diverse disease populations, including chronic neuropathic pain (Knop et al., 2001; Magbagbeola, 2001; McCormack, Horne & Sheather, 1988; Paul-Dauphin et al., 1999).

Nerve inflammation caused by virus infection triggers the ongoing neuralgia pain. Local and systemic inflammation is one of the causes and biomarkers underlining PHN pain. Interleukin-6 (IL-6) is an inflammatory mediator secreted by T cells and macrophages and is associated with central sensitization in PHN patients (Bayat et al., 2015). β-endorphin (β-EP) is an endogenous neuropeptide participating in pain signal transmissions by regulating the release of peripheral inflammatory factor such as substance P (SP) (Theoharides, 2017).

The current trail aimed to systematically evaluate the efficacy and safety of a combined therapy of PRF and NBT compared to traditional single-modality treatment such as oral drugs, nerve blocker administration, and PRF for PHN patients by recording VAS score in cotton-swab test. We also explored the underlying analgesic mechanisms by measuring the plasma IL-6, SP, and β-EP.

## Materials and Methods

### Study design and participants

**Figure 1:**
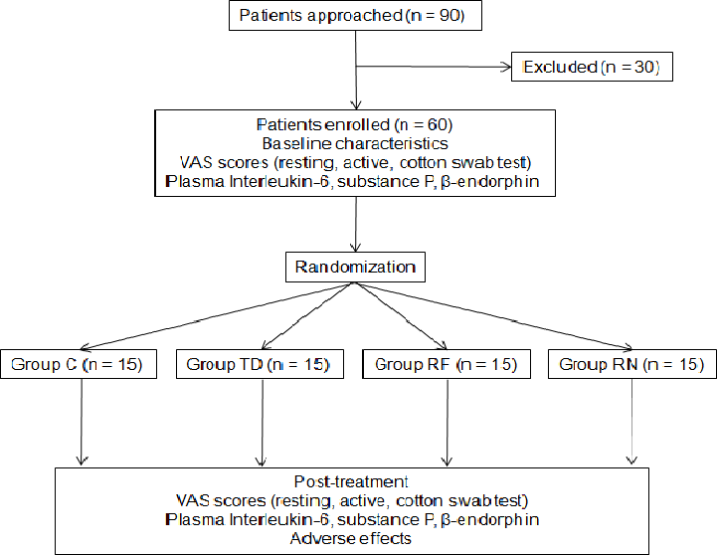
CONSORT flow diagram. Group C, drug control group (basic drugs, gabapentin capsules, tramadol hydrochloride). Group TD, nerve block therapy drug group. Group RF, pulsed radiofrequency group. Group RN, combined treatment group (pulsed radiofrequency combined with nerve block therapy.

This study was a prospective, randomized clinical trial, performed in an urban hospital in China. The study protocol was approved by the Medical Ethics Committee of Wuxi People’s Hospital (medical ethics number: 2016KYSL-01-01). This study was registered at the Chinese Ethics Committee of Registering Clinical Trials (ChiCTR-INR-17011094). A total of 90 patients who had PHN for more than 1 month were approached and 60 of them were enrolled and completed the study (Fig. 1). All participants gave verbal informed consent. Their ages ranged from 40 to 80 years and weight from 50 to 80 kg, with cardiac function grade I or II, and normal coagulation functions. Pregnant patients and those with severe liver and kidney dysfunctions, respiratory infections, or allergy to the test drug were excluded. Sixty PHN patients were divided into four groups according to a computer-generated random number table (*n* = 15 in each group): the conventional puncture group (group CP), the nerve block therapy group (group NB), the PRF group (group PRF), and the combined treatment group (PRF combined with NB therapy, group CT).

### Study protocol

After admission, routine examinations, including blood test for coagulation and hepatorenal function, were performed. All patients were given traditional oral drugs (gabapentin capsules 0.9 g/d, tramadol hydrochloride sustained release tablets 0.2 g/d) and all treatment procedures were performed in the operating room. Patients were placed in an appropriate position with electrocardiographic monitoring and oxygen inhalation (2 l/min). Patients in group CP received a simultaneous conventional puncture in the affected nerve. Patients in group NB received a 5-ml local drug injection in the affected nerve (mecobalamin injection, 1 mg/ml; extract from rabbit skin inflamed by vaccinia virus for injection, 6 ml; 1% ropivacaine, 2 ml; 0.9% normal saline, 11 ml). Vertebral nerve roots or nerves corresponding to the area of herpes zoster infection were identified if abnormal sensation occurred in the corresponding affected area (e.g., muscle twitching and abnormal temperature). Patients in group PRF were treated with three courses of PRF (Baylis PM230 PRF generator, 42 °C, 120 s). Patients in group CT received PRF treatment followed immediately by NBT. Patients in groups PRF and CT were punctured with Baliys PMF18-100-5 casing puncture needle, which was then connected to the PRF treatment instrument (Baylis PM230) probe. The test mode was 50 Hz, 0.3 V and the treatment mode was 42 °C, 120 s for three courses and the treatment interval was 1 min. After PRF treatment, 5 ml of the drug was injected for group CT patients. The puncture point was compressed to stop bleeding after the needle was removed. All patients returned to the ward after treatments followed by a 5–10 min observation period for stable vital signs and no complaints of discomfort.

### Measurements of outcome

A 4-ml blood sample was collected at admission (baseline), before the operation, and at 12 h after the operation. The plasma cytokine and neuropeptides were detected by enzyme-linked immunosorbent assay (ELISA) according to the manufacturer’s instructions: IL-6 (Dakota Biotechnology Co., Ltd., Beijing, China), β-EP (Abnova Company, Taipei, Taiwan), and SP (R & D Company, Minneapolis, MN, USA).

To access pain relief effect of various treatments, a clean cotton swab was used to brush the skin in the areas controlled by the affected nerves at a speed of 1–2 cm/s to induce pain and the VAS score was assessed at the admission (basic state), before the operation, and at 12 h after operation. VAS scores in the resting and active states were recorded as control. The VAS scores were interpreted as follows: 0, analgesia; 1–3, mild pain, which was bearable; 4–6, moderate pain, which could affect sleep; 7–10, severe, unbearable pain. The number of adverse events (nausea, vomiting, constipation, puncture point hemorrhage, swelling and redness) within 12 h after operations was also recorded.

### Statistical analysis

Data with normal distributions were presented as mean ± standard deviation. One-way ANOVA was used for comparisons among groups. Data with skewed distributions were presented as median with confidence intervals. The enumeration data were measured using the *χ*^2^ test. Rank data were compared by the rank sum test. The sample size was calculated based on the primary outcome analysis of comparisons of VAS scores by one-way ANOVA. With a moderate effect size of 0.25, alpha error of 0.05, and four groups of patients, a sample size of 280 patients was required to achieve a statistical power of 0.95 (GPower 3.1.9.2., USA). *P* < 0.05 was defined as statistically significant. The data were analyzed using SPSS 18.0 statistical software package (SPSS Inc., Chicago, IL, USA).

## Results

Here we enrolled and completed studies of total 60 PHN patients, of which 20 had chest and back pain, 35 had waist and back pain, and five had neck-shoulder-back pain (Fig. 1). There were no statistically significant differences in baseline characteristics of the patients among the four groups (Table 1). There was no significant difference in VAS scores before treatment compared with those at admission among the four groups (*p* >0.05). VAS scores in the resting and active states, and during cotton swab reactions decreased after each treatment. After enrollment, treatments including cervical and thoracolumbar paravertebral PRF therapy and NBT were performed accordingly. The most significant VAS score decrease was observed in group CT (*P* < 0.001), as shown in Table 2.

**Table 1:** Baseline characteristics among different groups (*N* = 15 in each group).

Items	Group C	Group TD	Group RF	Group RN
Age (year, mean ± SD)	66 ± 5	68 ± 7	69 ± 5	65 ± 6
Weight (kg, mean ± SD)	64 ± 8	62 ± 10	61 ± 7	63 ± 9
Height (cm, mean ± SD)	162 ± 8	159 ± 9	163 ± 8	160 ± 9

**Notes.**

Group C, drug control group (basic drugs, gabapentine capsules, tramadol hydrochloride).

Group TD, nerve block therapy drug group.

Group RF, pulsed radiofrequency group.

Group RN, combined treatment group (pulsed radiofrequency combined with nerve block therapy.

**Table 2:** Comparison of VAS scores in the resting and active states and during cotton swab reaction among various groups (*N* = 15).

Group	VAS score in resting state		VAS score in active state		Cotton swab induced VAS score	
	At admission	After treatment	At admission	After treatment	At admission	After treatment
C	7.73 ± 0.88	4.80 ± 0.77	8.80 ± 0.86	5.27 ± 0.70	9.00 ± 0.76	4.73 ± 0.80
TD	8.07 ± 0.96	3.40 ± 0.63^a^	8.80 ± 0.0.86	4.07 ± 0.59	9.07 ± 0.80	3.60 ± 0.51^a^
RF	7.80 ± 0.94	3.07 ± 0.59^ab^	8.80 ± 0.86	3.53 ± 0.74^ab^	8.80 ± 0.86	3.20 ± 0.67^ab^
RN	7.80 ± 0.86	1.87 ± 0.52^abc^	8.67 ± 0.82	2.27 ± 0.46^abc^	8.80 ± 0.86	3.20 ± 0.65^abc^

**Notes.**

Group C, drug control group (basic drugs, gabapentine capsules, tramadol hydrochloride).

Group TD, nerve block therapy drug group.

Group RF, pulsed radiofrequency group.

Group RN, combined treatment group (pulsed radiofrequency combined with nerve block therapy).

Compared with group C, ^a^*P* < 0.001; compared with group TD, ^b^*P* < 0.001; compared with group RF, ^c^*P* < 0.001.

The plasma levels of IL-6, SP, and β-EP were not significantly different compared to those basic level at admission among groups NB, PRF, and CT (*p* < 0.05). The plasma IL-6 and SP decreased (*p* < 0.001) and plasma β-EP increased (*p* < 0.001) in each group after treatment. After treatment, the plasma IL-6 and SP were significantly lower (*p* < 0.001), while those of β-EP were significantly higher (*p* < 0.001) in groups NB, PRF, and CT, compared to those of group CP. The most significant increase of β-EP levels was observed in group CT (Table 3).

**Table 3:** Comparison of plasma level of IL-6,SP and *β*-EP among various groups (*N* = 15).

Group	IL-6		SP		*β*-endorphin	
	At admission	After treatment	At admission	After treatment	At admission	After treatment
C	138.45 ± 6.04	90.99 ± 4.28	364.40 ± 34.82	234.34 ± 27.69	125.67 ± 24.98	201.24 ± 36.43
TD	136.15 ± 6.34	61.67 ± 4.09^a^	347.67 ± 40.39	153.33 ± 14.58^a^	127.13 ± 19.43	245.73 ± 24.62^a^
RF	134.87 ± 7.16	57.17 ± 4.28^ab^	355.93 ± 31.62	134.48 ± 16.16^ab^	129.44 ± 14.98	276.09 ± 18.32^ab^
RN	136.63 ± 6.26	41.02. ± 4.00^abc^	361.23 ± 23.73	114.33 ± 4.17^abc^	123.19 ± 20.07	326.20 ± 40.62^abc^

**Notes.**

Group C, drug control group (basic drugs, gabapentine capsules, tramadol hydrochloride).

Group TD, nerve block therapy drug group.

Group RF, pulsed radiofrequency group.

Group RN, combined treatment group (pulsed radiofrequency combined with nerve block therapy.

After treatment, each group was compared with group C, ^a^*P* < 0.001; compared with group TD, ^b^*P* < 0.001; compared with group RF, ^c^*P* < 0.001.

There were no significant differences in the number of adverse events among the four groups (*p* > 0.05) and no incidence of swelling, redness, and bleeding in puncture sites within 12 h after the operation (Table 4).

**Table 4:** Comparison of adverse reactions occurred in each group (*N* = 15).

Items	Group C	Group TD	Group RF	Group RN
Nausea and vomiting	2 cases	1 case	2 cases	1 case
Constipation	1 case	1 case	1 case	1 case

**Notes.**

Group C, drug control group (basic drugs, gabapentine capsules, tramadol hydrochloride).

Group TD, nerve block therapy drug group.

Group RF, pulsed radiofrequency group.

Group RN, combined treatment group (pulsed radiofrequency combined with nerve block therapy.

## Discussion

Postherpetic neuralgia is a common neuropathic pain that can seriously affect sleep and life quality of the patients. The main characteristics of PHN include spontaneous pain, paroxysmal electrical shock-like pain or acupuncture-like pain, hyperalgesia, and sensory loss. Once diagnosed, treatment regimen should be aimed at pain controlling and reducing adverse events related to treatments. To measure the extent of remission of the pain, the dynamic mechanical cotton swab reaction was used in this study (Jensen & Finnerup, 2014). At present, although there is no single treatment for all PHN patients (Werner et al., 2017), oral administration of gabapentin and tramadol sustained-release-tablets are commonly used as basic treatments. PRF and NB therapy are safe and effective options (Malec-Milewska et al., 2014; Wan et al., 2016). Applying bipolar high-voltage PRF treatment on the dorsal root ganglion in the affected area for a prolonged time effectively could relieve pain, reduce drug dosage, and improve the life quality of the patients (Saxena et al., 2016). On the other hand, NB treatment can effectively alleviate nerve cell edema and improve the peripheral blood circulation using cortisol glucocorticoids, low-dose ropivacaine, and the neurotrophic and anesthetic drugs (Malec-Milewska et al., 2014).

Our study showed that VAS scores significantly decreased after treatment, in the resting and active states and during cotton swab reaction in all 4 group patients, with the most significant decrease observed in group CT, suggesting that oral drugs, NBT, and PRF relieve pain and hyperalgesia in PHN patients. PRF was more effective in pain relief than NBT, and a combination of PRF and NBT was more effective than either PRF or NBT alone. In addition, there were no significant differences in nausea, vomiting, and constipation among the four groups of PHN patients. There was no incidence of swelling, redness, bleeding, or infection in the puncture points too. Taken together, our data suggest that the combined treatment of PRF with NBT is safe and effective for PHN patients.

To understand the mechanism of our treatments, plasma cytokine and neuropeptides in PHN patients were measured. Increased inflammatory cytokine IL-6 in PHN patient plasma sensitizes central sensory and highly correlates with hyperalgesia (Bayat et al., 2015). However, pituitary gland secreted neuropeptide β-EP significantly decreased in the cerebrospinal fluid of PHN patients (Zhao et al., 2017). β-EP directly inhibit pain transduction by activating opioid receptors in the pain-related regions in the brain and spinal cord. The plasma level of β-EP is highly correlated with the analgesic effect of opioids, which inhibit the synthesis of the nociceptive neuropeptide SP (Bruehl et al., 2012). In our study, the plasma levels of IL-6 and SP decreased and those of β-EP significantly increased in all treatment groups, suggesting that all treatments, including oral drugs, NBT, and PRF, could reduce the release of inflammatory mediators, inhibit central sensitization, and regulate the immunoreactivity expressions. Such reduction effect was most obvious in the combined therapy group, in consistent with our observation that PRF together with NBT relieves pain superior to either PRF or NBT alone. This synergistic effect of PRF and NBT was also observed in chronic cervical radicular pain (Wang et al., 2017; Xiao et al., 2015), suggesting a general potential application of this combination therapy in chronic pain management.

In this study, we performed a randomized, parallel-group study in 60 outpatients to evaluate the efficacy and safety of four different treatment regimens. Limitations of the current study include its design as a single-center study and its small sample size. We did not assess the effects of liver or renal dysfunction on the treatment effects either. Sample size is an important factor to detect a statistically significant difference (Sullivan & Feinn, 2012). Our study might have limited statistical power to detect difference among the groups. Future multi-center studies with larger sample sizes are warranted to validate our findings.

## Conclusions

In conclusion, PRF combined with NBT could safely and effectively relieve pain and hyperalgesia in PHN patients by up-regulating the expression of β-EP and reducing the levels of IL-6 and SP in plasma.

##  Supplemental Information

10.7717/peerj.4852/supp-1 Supplemental Information 1 CONSORT checklist

10.7717/peerj.4852/supp-2 Supplemental Information 2 Protocol

10.7717/peerj.4852/supp-3 Supplemental Information 3 Raw data

## Abbreviations

PHN: Postherpetic neuralgia
NBT: nerve block therapy
PRF: pulsed radiofrequency
VAS: visual analogue scale
IL-6: interleukin-6
SP: substance P
β-EP: β-endorphin
ELISA: enzyme-linked immunosorbent assay

## Additional Information and Declarations

### Data Availability

The following information was supplied regarding data availability: The raw data are provided in a Supplemental File.

### Clinical Trial Registration

The following information was supplied regarding Clinical Trial registration: Chinese Clinical Trial Registry: chiCTR-IPQ-17011094.
