# Supplementary material for: Combined therapy of pulsed radiofrequency and nerve block in postherpetic neuralgia patients: a randomized clinical trial
Source: PeerJ. 2018 Jun 4;6:e4852. doi: 10.7717/peerj.4852 (PMC5991296; doi:10.7717/peerj.4852)
Supplement: Supplemental Information 2 [file peerj-06-4852-s002.pdf]

Summary of protocol:

The postherpetic neuralgia (PHN) is the most common chronic neuropathic pain caused by viral infection. In clinical routine treatment, only a few patients can achieve a reduction in pain by more than 50%. Early use of nerve block therapy (NBT) can effectively shorten the duration of herpes zoster pain; the pulsed radiofrequency (PRF) can regulate the function of the affected nerves to safely and effectively relieve the PHN pain, but the specific analgesic mechanisms of both treatment methods and the combined effect are not clear yet. IL-6 is an inflammatory factor secreted by T cells and macrophages and is associated with the central sensitization in PHN patients ; β-endorphin (β-EP) is an endogenous neuropeptide , and it can participate in the transmission of pain signals through regulating the release of peripheral inflammatory factor , pain factor and substance p (SP). This study was to observe the effect of combined application of oral drug, nerve block and pulsed radiofrequency in the treatment of postherpetic neuralgia and explore the potential mechanism of PHN treatment, provide reference for clinical treatment.

Study objective:

To observe the effect of combined application of oral drug, nerve block and pulsed radiofrequency in the treatment of postherpetic neuralgia and explore the potential underlying mechanisms.

Study design:

Prospective randomized clinical trial

Interventions:

Drug control group, nerve block therapy, pulsed radiofrequency, and the combined treatment

Sample size:

60

Outcomes:

plasma levels of IL-6, SP and β-EP

VAS scores

side effects

Participant selection and enrollment:

Subjects will be recruited by:

Research associate

Inclusion criteria:

Aged 40 to 80 years and weighted 50 to 80kg, with cardiac function grade Ⅰ and Ⅱ and without abnormal coagulation function

Exclusion criteria:

Severe liver and kidney dysfunction, respiratory infections, related drug allergy or pregnancy

Summary of study procedure:

After admission, the routine examinations such as coagulation function, hepatorenal function and blood routine were performed, the patients in various groups were treated with conventional oral drugs such as basic drugs (gabapentine capsules 0.9g / d, tramadol hydrochloride sustained release tablets 0.2 g / d). The patients in group C received basic drug treatment to relieve pain and simultaneously underwent conventional puncture for the affected nerve, and they didn’t receive drug injection and PRF treatment; the patients in group TD were given 5ml locally injected drugs for the affected nerve (mecobalamin injection 1mg / 1ml, extract from rabbit skin

inflamed by vaccinia virus for injection 6ml, 1% ropivacaine 2ml, 0.9% normal saline 11ml); the affected nerves of patients in group RF were treated with PRF (Baylis PM230 pulsed radiofrequency generator, 42 ℃, 120s) for 3 courses. The patients in group RN received NBT immediately after PRF treatment.

All patients who needed to receive treatment operations should enter into the operating room for operative treatment, the vertebral nerve roots or nerves corresponding to the area of herpes zoster infection were selected and located. Thereafter, ECG monitoring and conventional oxygen inhalation (2L / min) were carried out and the patient was placed in an appropriate position, subsequently routine disinfection and draping were performed. The patients in group RN and RF were punctured with Baliys PMF18-100-5 casing puncture needle, which was then connected to the pulse radio frequency treatment instrument (Baylis PM230) probe , the test mode was adjusted（50HZ，0.3V）. Location was performed if abnormal sensation occurred in the corresponding affected area (muscle twitching, abnormal temperature, etc.), and then the mode was adjusted as the treatment mode (42 ℃, 120s) for 3 courses and the interval between treatments was 1min, subsequently 5ml drug was injected after treatment. After the operation is completed, the puncture point was compressed to stop bleeding, and the patient was observed for 5-10min. If the vital signs were stable and no discomfort was felt, the patient could return to the ward.

Outcome measurements

4 ml blood sample from elbow vein was collected at admission (basic state), before operation and at 12h after operation, and serum indexes were detected by ELISA. The upper layer of serum was collected after centrifugation at 3000rpm for 5 min. The levels of serum cytokines IL-6 (Dakota Biotechnology Co., Ltd., China), β-EP (Abnova Company, China) and SP (R & D Company, USA) were detected. Steps: the reagent kit was stored for 20-30mmin for a balance at room temperature, simultaneously the standard substance and Washing buffer were prepared. 100 μl of serum was added into the sample well of IL-6. 50 μl of serum was added respectively into the sample wells of the β-EP and SP. The plate was incubated at room temperature for 1 h and washed 3 times with Washing buffer for 1 min each time. Thereafter, the plate was added with the detection antibodies and incubated at room temperature for 1h, subsequently it was washed 3 times with Washing buffer for 1min each time. The plate was added with the substrate and incubated in the dark for 15-30 min, and then added with 50 μl of stop buffer to terminate

the reaction. The absorbance was measured by a microplate reader at a wavelength of 450 nm. The standard curves were drawn and the concentrations of samples were calculated according to the curves.

VAS scores respectively in the resting and active states and during cotton swab reaction were recorded. the number of cases with adverse effects (nausea, vomiting, constipation, puncture point hemorrhage, swelling and redness of drugs and operations within 12h was recorded. VAS scores: 0 point indicated analgesia; 1-3 points indicated mild pain, which was bearable; 4-6 points indicated moderate pain, which could affected sleep; 7-10 points indicated severe pain, which was unbearable. Cotton swab reaction: the degree of pain in the patients induced by the swab touch was detected. A clean cotton swab was used to brush the skins in the areas controlled by the affected nerves of the patients at a speed of 1-2cm/s to induce pain and VAS score was assessed.

Participant confidentiality:

All data collection instruments are kept in in a secured manner and password protection of all electronic data files will be performed. The hard copies of the data collection forms will be kept in a secured manner in the office of the PI in the Department of Orthopedics. Access to these documents will be restricted to study personnel. Data will be deidentified after completion of the study.

Anticipated risks or adverse events:

Nausea, vomiting, constipation, puncture point hemorrhage, swelling and redness

Adverse events reporting:

The principal investigators will be primarily responsible for monitoring the safety and efficacy of the study, executing the data safety monitoring plan, and complying with all reporting requirements to the ethics committee. The principal investigators will review the approved

protocol prior to enrolling research participants and perform regular review of study progress and patient outcomes on a weekly basis.
